# Safe Oral Triiodo-L-Thyronine Therapy Protects from Post-Infarct Cardiac Dysfunction and Arrhythmias without Cardiovascular Adverse Effects

**DOI:** 10.1371/journal.pone.0151413

**Published:** 2016-03-16

**Authors:** Viswanathan Rajagopalan, Youhua Zhang, Kaie Ojamaa, Yue-feng Chen, Alessandro Pingitore, Christine J. Pol, Debra Saunders, Krithika Balasubramanian, Rheal A. Towner, A. Martin Gerdes

**Affiliations:** 1 Department of Biomedical Sciences, New York Institute of Technology-College of Osteopathic Medicine, Old Westbury, New York, United States of America; 2 Feinstein Institute for Medical Research, Manhasset, New York, United States of America; 3 CNR Clinical Physiology Institute, Pisa, Italy; 4 Oklahoma Medical Research Foundation, Oklahoma City, Oklahoma, United States of America; David Geffen School of Medicine at UCLA, UNITED STATES

## Abstract

**Background:**

A large body of evidence suggests that thyroid hormones (THs) are beneficial for the treatment of cardiovascular disorders. We have shown that 3 days of triiodo-L-thyronine (T3) treatment in myocardial infarction (MI) rats increased left ventricular (LV) contractility and decreased myocyte apoptosis. However, no clinically translatable protocol is established for T3 treatment of ischemic heart disease. We hypothesized that low-dose oral T3 will offer safe therapeutic benefits in MI.

**Methods and Results:**

Adult female rats underwent left coronary artery ligation or sham surgeries. T3 (~6 μg/kg/day) was available in drinking water ad libitum immediately following MI and continuing for 2 month(s) (mo). Compared to vehicle-treated MI, the oral T3-treated MI group at 2 mo had markedly improved anesthetized Magnetic Resonance Imaging-based LV ejection fraction and volumes without significant negative changes in heart rate, serum TH levels or heart weight, indicating safe therapy. Remarkably, T3 decreased the incidence of inducible atrial tachyarrhythmias by 88% and improved remodeling. These were accompanied by restoration of gene expression involving several key pathways including thyroid, ion channels, fibrosis, sympathetic, mitochondria and autophagy.

**Conclusions:**

Low-dose oral T3 dramatically improved post-MI cardiac performance, decreased atrial arrhythmias and cardiac remodeling, and reversed many adverse changes in gene expression with no observable negative effects. This study also provides a safe and effective treatment/monitoring protocol that should readily translate to humans.

## Introduction

Thyroid hormones (THs) serve as a master regulator in the control of diverse molecular, physiological and pathophysiological remodeling processes in the heart and vascular system [1, 2]. Studies have repeatedly shown increased morbidity and mortality in heart failure (HF) patients with low serum TH levels [3, 4]. In addition, irrespective of the serum thyroid status, peripheral thyroid signaling mechanisms are impaired in models of cardiovascular disorders including increased deiodinase 3 enzymes [5]. THs regulate expression of myosin heavy chains (MHC) and several studies have demonstrated that TH treatment reversed ‘*fetal gene reprogramming or re-expression*’ with corresponding improvement in functional performance [6–8].

Many have shown that THs can be safely administered in human HF with significant short-term therapeutic benefits [9–11]. Nonetheless, the impetus for developing TH analogs to treat HF is largely based on the premise that THs are too dangerous for such use, implying that a therapeutic window may not exist. Ironically, current opinions on this topic have been strongly influenced by two clinical trials involving TH analogs, rather than actual THs, that showed adverse effects from doses that appeared to be excessive [12, 13]. Recently, we have successfully demonstrated prevention/attenuation of left ventricular (LV) dysfunction/remodeling without significantly altering thyroid homeostasis using low dose oral triiodo-L-thyronine (T3) treatment in models of diabetic cardiomyopathy and hypertensive heart failure [5, 14]. The major goal of this study was to explore a safe therapeutic window for oral T3 treatment of myocardial infarction (MI) in rats and verify efficacy of a selected dose within that window. The ongoing THiRST (Thyroid Hormone Replacement Therapy in ST Elevation MI) trial investigating oral T3 treatment of STEMI patients served as a major catalyst for this animal study (http://www.ponte-project.eu/).

We have previously demonstrated that 3 days of T3 treatment in rats following MI resulted in decreased myocyte apoptosis and increased LV contractility [15]. We hypothesized that our therapeutic T3 treatment/monitoring protocol will achieve safe cardioprotective effects in rats following MI. T3 was chosen instead of thyroxine (T4) since the conversion mechanisms of T4 into T3 is known to be impaired in nonthyroidal illness syndrome [16]. The administration of T3 largely by-passes this potential problem of bioavailability by direct application of the active form of TH [17]. This study, for the first time, has thus established a safe therapeutic window for low-dose oral T3 administration with marked improvements in cardiac structure and function, rhythm, remodeling and gene expression without adverse cardiovascular effects. This also serves as a translatable TH protocol for MI that provides much needed guidance for clinical trials, as in subclinical thyroid dysfunction.

## Methods

### Experimental design

This study was approved by the Institutional Animal Care and Use Committees at Oklahoma Medical Research Foundation (OMRF) and New York Institute of Technology College of Osteopathic Medicine (NYIT-COM) and is in compliance with the “Guide for the Care and Use of Laboratory Animals” (National Institutes of Health Publication No. 85–23, Revised 1996) and presented based on Animal Research: Reporting of *In Vivo* Experiments, ARRIVE guidelines (National Centre for the Replacement Refinement & Reduction of Animals in Research, NC3Rs). Adult female Sprague–Dawley rats (Harlan Labs) aged 11 weeks old were used in this study. Following acclimatization, the rats were examined to exclude any pre-existing conditions that may complicate surgical outcome. Neck and chest fur were shaven and disinfected with betadine and 70% alcohol. The chest was draped to maintain asepsis and the surgical tools were sterilized with hot bead sterilizer before and between surgeries. As described previously [18], following standard intraperitoneal ketamine/xylazine anesthesia and endotracheal intubation and ventilation (~70 breaths per minute), MI was produced by ligation of the left anterior descending coronary artery by the same microsurgeon (YFC) at NYIT-COM and OMRF. Following completion of the microsurgical procedure, the chest retractor was removed and the ribs were drawn together using continuous 5–0 silk sutures. The tidal volume (1 ml per 100 gram body weight) was temporarily increased to expel air from the chest, and skin was closed with continuous 5–0 sutures.

Immediately following surgery, MI survivors were *randomly* assigned to an MI group (n = 22) and an MI+T3 group (n = 24). A Sham group (n = 18) was produced with a similar procedure except that the suture was tied loosely around the coronary artery. These animal counts include surgeries performed at both the institutes (NYIT-COM and OMRF). To ensure sufficient number of rats were available despite any potential immediate-post-op mortality, we operated on extra rats in the MI groups. Following extubation, intramuscular Buprenorphine analgesia was administered. T3 was dissolved in ethanol/glycerol and added to drinking water (0.05–0.08 μg/ml) for a total duration of 2 months. This provides a dose of 5–8 μg/kg/d based on daily water consumption (~25 ml/day from pilot studies) and serum TH feedback inhibition response from previous studies [5]. Vehicle was used in rats not treated with T3 using the aforementioned diluents. The dose range was selected based on a pilot study showing that heart rate, heart weight and water consumption begins to increase in some rats at doses above 8 μg/kg/d.

At the NYIT-COM site, two-dimensional echocardiograms (GE Vivid 7 Dimension, Horten, Norway; M12L transducer) were obtained from LV short-axis to visualize the extent of infarction immediate post-op [18]. Animals that developed small infarcts [19] and those that did not survive for more than 48 hours were excluded (data not shown). At 2 months post-MI, *blinded*, *standard isoflurane-anesthetized* (3% induction, 1.5% maintenance) cardiac catheterization studies were followed by body weight measurements and terminal experiments (surgical plane isoflurane anesthesia)–blood collection for serum TH levels and tissue collection for histology (fixed in paraformaldehyde) and molecular studies (stored at -80°C). Cardiac function and remodeling were also assessed by Magnetic Resonance Imaging (MRI) at about 1 week, 1 month, and 2 months of T3 treatment at the OMRF site from another cohort followed by terminal experiment protocols as outlined above. The MRI acquisition was performed at OMRF and all animals (1 to 3 per cage) were kept on a 12-hour light/dark cycle and food and water were provided *ad libitum*. Nine rats became ill or died in the immediate post-op period prior to experimental endpoint. Animals exhibiting symptoms indicative of severe illness/moribundity were humanely euthanized per the IACUC protocol.

### Determination of LV Volumes and Function by Cardiac Magnetic Resonance Imaging

For MRI studies at the OMRF site, rats were anesthetized with inhaled isoflurane (1.5% at 1 L/min oxygen flow) via a nose cone. During the experiment, the rats were positioned supine on a nonmagnetic warming pad to maintain constant body temperature throughout the MRI study. Experiments were performed on a 7 Tesla MRI scanner (Bruker BioSpin, Ettlingen, Germany) as described [20]. For imaging an IntraGateFLASH sequence with *t*e = 2.1 ms, *t*r = 10 ms, and 300 repetitions was used for all experiments. The heart was initially positioned by measurement to the center of the magnet and then further set by using fast imaging with steady state precession as the rat was moved. Single slice images in all three planes (axial, sagittal and coronal) were acquired using an IntraGateFLASH sequence with *t*e = 2.1 ms and *t*r = 10 ms. For cine and volume imaging a field of view of 6.00 × 6.00 cm was chosen and 256 each of phase encode and read steps were used to resolve the spatial distribution of excited spins.

All MRI data are adjusted to accommodate for rate changes and the data are reflective of the ambient cardiac function and rate. The cardiac period (time between the peaks of the *R* wave in the electrocardiogram, ECG trace) ranged from ~220–280 ms, and a respiratory rate of ~18–30 bpm. The images at each increment of the period were combined and displayed by cine in the mid-ventricular short axis slice. These provided data for assessment of LV systolic and diastolic dynamics, and calculations of fractional shortening and wall thickness. For volume calculations, a new set of images were collected with the slice position advanced by the slice thickness of 1.0 mm.

For LV blood volume measurements, endocardial borders were manually delineated. The total LV volume at end diastole and end systole was estimated by taking the sum of all cavity slice volumes assuming a uniform thickness of excitation across a chosen slice at the two trigger points. For assessment of LV systolic and diastolic dynamics, the cavity slice volume was measured in all acquired images and was plotted against the time from onset of the QRS trigger, and a volume-time curve was established.

### Atrial arrhythmia inducibility and hemodynamic measurements

Electrophysiological studies were performed at the NYIT-COM site on rats operated by the same microsurgeon based on Zhang et al [21]. Under anesthesia, a 1.6F octopolar Millar electrophysiology catheter (EPR-802, Millar Instruments, Inc., Houston, Texas) was inserted through right jugular vein and advanced into the right atrium with 8 poles recording atrial electrograms. Standard surface ECG lead II and 3 right atrial electrocardiograms from 3 pairs of electrodes were displayed and recorded using a PowerLab data acquisition system (ADInstruments, Colorado Springs, CO). The purpose of recording 3 atrial electrograms from distal, middle, and proximal pairs was to facilitate determination of atrial capturing and arrhythmia pattern. Poles 5 and 6 (the third pairs counted from catheter tip) were used for pacing. Regular pacing and standard S1S2 pacing protocols were used. Burst pacing containing 200 impulses at 50 Hz was used to induce atrial tachyarrhythmias (tachycardia or fibrillation; ATA). ATA was defined as rapid atrial activations with varying electrogram morphology lasting ≥0.5 seconds. The duration of the subsequent spontaneous ATA after burst pacing was documented. The mean ATA duration based on 5 such tests was used to reflect the ATA substrate in each animal. If induced spontaneous ATA lasted more than 5 minutes, ATA was considered long-lasting.

LV hemodynamics was obtained under anesthesia by catheterization through the right carotid artery using a 1.9F SciSense pressure-volume catheter (Transonic Scisense Inc., London, Ontario, Canada). The tip of the catheter was advanced through the aorta into the left ventricle to study heart rate, LV and arterial pressures and change in pressure over time (dP/dt). The data were acquired and analyzed by the LabScribe software (iWorx Systems, Inc., Dover, NH).

### Serum hormone measurements

The collected blood was centrifuged at 4000 rpm for 15 minutes at 4 deg C. The supernatant was aliquoted and stored at -80° Celsius until assayed. Serum T3 and T4 levels were measured using Enzyme-linked immune-sorbent assay (ELISA) kits according to the manufacturers’ specification. T3 and T4 kits were obtained from Monobind Inc. (Lake Forest, CA). The kits have been shown to produce excellent results in rats in our laboratory. Rat thyroid stimulating hormone (TSH) was assayed using a kit from ALPCO (Salem, NH).

### Tissue collection and histology

During terminal experiments, the chest was opened and the heart was arrested in diastole by injection of 0.2 M potassium chloride in phosphate buffered saline (PBS) via the LV apex. Blood was collected from the ventricles and processed as described below. The heart was then rapidly removed and cannulated through the aorta with an 18 gauge gavage needle to allow coronary perfusion with ice cold 0.2% 2, 3- butanedione monoxime dissolved in PBS. Fat and aorta were then trimmed off and heart and chamber weights were documented quickly. Basal and apical LV portions were stored at -80C and used for molecular analyses. The transverse mid-slice of the LV with septum was used for histological analyses. Paraformaldehyde-fixed 5 μm thick LV tissue sections were stained with Masson’s Trichrome stain at the Histology laboratory of the NYIT-COM. Images were acquired using Olympus BX53 microscopes and transmural infarct tissue area dimensions were analyzed using Image-Pro plus software (Media Cybernetics, Bethesda, MD).

### Real-time quantitative PCR for gene expression

RNA was isolated using TRIzol reagent followed by RNA purification using PureLink RNA mini kit and DNaseI kit (Invitrogen, Carlsbad, CA) as previously described [22]. RNA quantity and quality were determined and validated using a NanoDrop 1000 (Thermo Scientific, Wilmington, DE) and Agilent 2100 Bioanalyzer (Agilent Technologies, Santa Clara, CA). Equal amounts of RNA from each sample were converted to cDNA using RT^2^ First Strand Kit (Qiagen Inc., Valencia, CA). Gene expression was evaluated by a custom-designed primer plate for more then 90 select genes that are known or predicted to be involved in thyroid signaling, MI, or HF using SYBR green/ROX detection (Qiagen Inc., Valencia, CA) and Roche Light Cycler 480. For the purposes of brevity, only those that are more closely relevant/significant are shown. *Ppia* (cyclophilin A) and *Rplp1* (ribosomal protein, large, P1) were used as housekeeping control genes and expression data was analysed using SABiosciences expression analysis online software (Qiagen Inc., Valencia, CA).

### Statistical analysis

All data are expressed as means±standard error unless otherwise noted with standard deviation and were compared using a two-tailed student’s t-test or one-way analysis of variance (ANOVA) with Newman-Keuls post-hoc test (GraphPad Prism) to examine significant differences between groups. Fisher exact test was used to compare the incidence of atrial tachyarrhythmias (ATA). The ATA duration data are expressed as median, first and third quartile (Q1–Q3) values delimiting bottom 25% and top 25% of the distribution and the extremes [21]. A nonparametric Kruskal–Wallis test followed by Dunn’s Multiple Comparison Test was used to compare the ATA duration data. A value of P < 0.05 was considered statistically significant.

## Results

### Morphometrics

Approximately 85% of rats undergoing MI surgery survived with large infarcts as visualized by echocardiogram or MRI and comprised the study population. This corresponds to a consistent 30%-50% infarction in both MI groups. The body weights were not altered by T3 (Table 1). Following MI, heart weight, LV+septal weight and both their ratios with respect to body weight were significantly increased indicating hypertrophy. T3 did not significantly increase these parameters indicating that the low-dose did not induce hypertophic changes.

**Table 1:** Morphometric data.

	Sham	MI	MI+T3
**Body weight (g)**	251±12	264±16*	260±17
**HW (mg)**	890±15	1213±29**	1212±28***
**LVW+SepW (mg)**	620±59	750±63***	690±71**§§
**RVW (mg)**	170±28	250±12	240±11
**HW/BW (mg/g)**	3.6±0.6	4.6±0.9**	4.7±0.98**
**(LVW+SepW)/BW (mg/g)**	2.5±0.2	2.8±0.2***	2.7±0.2*§
**RVW/BW (mg/g)**	0.7±0.1	0.9±0.4	0.9±0.4

Values are means±SD

MI–Myocardial Infarction; Triiodo-L-thyronine (T3)

BW–Body weight

HW–Heart weight

LVW+SepW–Left ventricular Weight including septal weight

RVW–Right ventricular weight

n = 13–16 sham; 20–21 MI+Veh; 22–24 MI+T3

*p<0.05 vs. Sham

**p<0.01 vs. Sham

***p<0.01 vs. Sham

^§^p<0.05

^§§^p<0.01 vs. MI.

### Serum thyroid hormone levels are unaffected following T3 treatment

**Fig 1:**
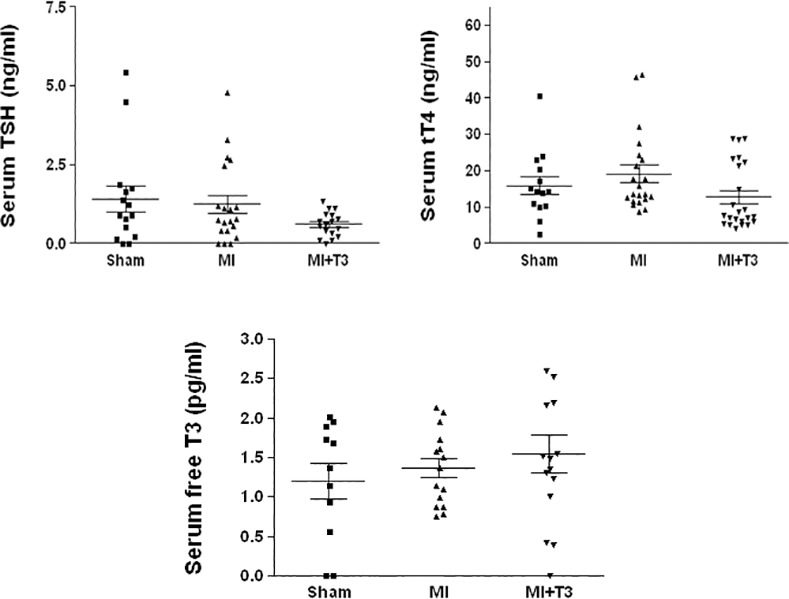
Serum thyroid hormone levels. Serum levels showed anticipated mild feedback inhibition inT4 and TSH without significant increase in free T3 levels. tT4–Total T4; TSH–Thyroid-stimulating hormone; T3–triiodo-L-thyronine; MI–Myocardial Infarction; tT4: n = 14 sham; 21 MI; 23 MI+T3; TSH: n = 15 sham; 20 MI; 17 MI+T3; free T3: n = 11 sham; 15 MI; 15 MI+T3.

In a separate cohort of rats using the same treatment protocol, we had performed serum total T4 (tT4) and TSH assays 2 weeks following sham/MI surgery. Although there were slight decreases following T3 treatment, they were not statistically significant (unpublished). Therefore, we did not repeat the 2-wk time-point in this study. Following 2-mo. T3 treatment in the current cohort, compared to MI, both serum tT4 and TSH showed a non-significant tendency to decrease with T3 –i.e., expected feedback inhibition responses following T3 treatment (Fig 1). Importantly, as intended, levels of the active form of TH (free T3) were not significantly increased above normal.

### Oral T3 treatment significantly improves MRI-based cardiac structure and function

**Fig 2:**
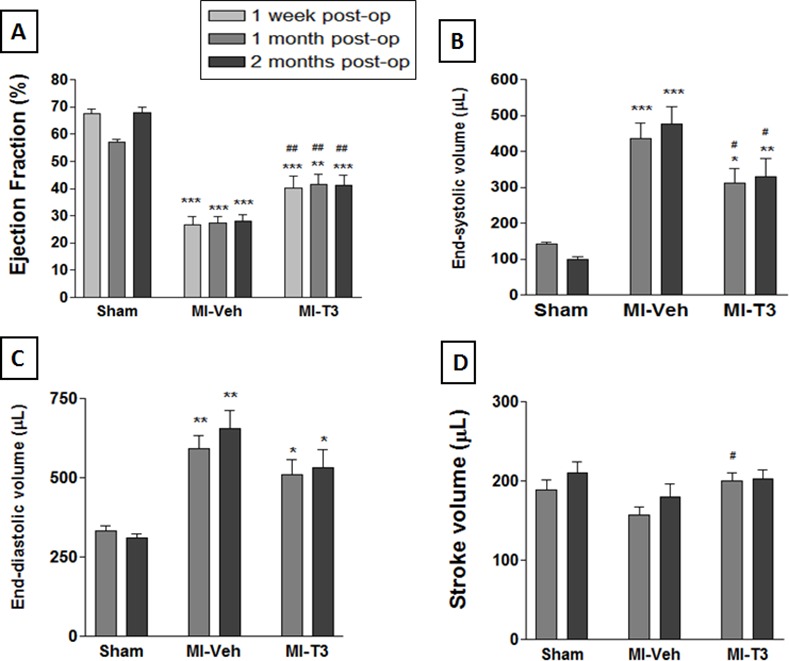
Magnetic Resonance Imaging shows significant improvement in LV function with T3 treatment. Compared to MI-Veh, oral T3 administration markedly improved MRI-based ejection fraction (A); EF. End-systolic (B), end-diastolic (C) and stroke volumes (D) also improved. * p<0.05, ** p<0.01, *** p<0.001 vs. Sham at corresponding time-point; # p<0.05, ## p<0.01 vs. MI-Veh at corresponding time-point; n = 8 Sham, 10 MI-Veh, 10 MI-T3 at 1 week; n = 6 Sham, 10 MI-Veh, 10 MI-T3 at 1 mo; n = 6 Sham, 10 MI-Veh, 10 MI-T3 at 2 mo.

Compared to the vehicle-treated MI rats, T3-treated MI rats exhibited marked and consistent improvement in ejection fraction (EF) by 2-mo (Fig 2 and S1 Fig). The increase in EF corresponds to 51% at 1 week (Wk), 52% at 1 Mo and 47% at 2 Mo post-MI. The end-systolic volume significantly decreased following T3 by 29% at 1 Mo and 31% at 2 Mo post-MI (p<0.05). These data indicate that T3 prevents deterioration of cardiac function following MI and improves cardiac geometry. Similar and sustained improvements in structure/function were noted at the earlier time points and additional comprehensive data were collected at 2-mo time-point.

### Oral T3 treatment reduces atrial tachyarrhythmia inducibility and improves LV contractility

**Fig 3:**
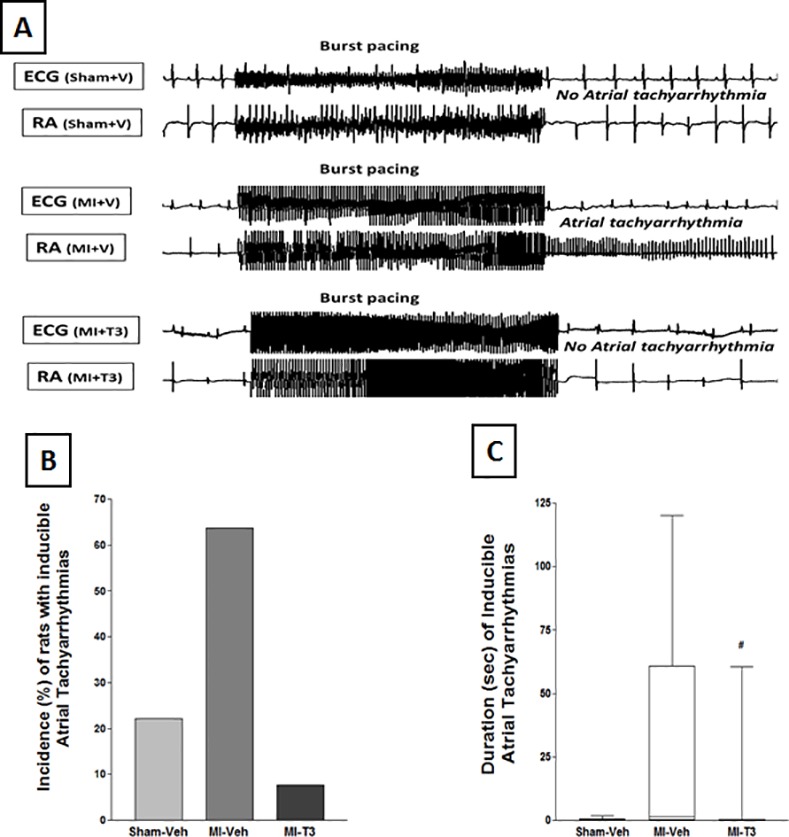
Oral T3 reduced duration and incidence of atrial tachyarrhythmias (ATA). The upper panel (A) shows representative traces of original electrocardiogram and right atrial (RA) electrograms after burst/tachypacing. This demonstrates rapid irregular atrial activations with varying electrogram morphology following MI, with T3 treatment preventing the effect. Importantly, the (B) incidence of ATA that persisted following discontinuation of experimental atrial burst/tachypacing was significantly diminished by 88% subsequent to T3 treatment post-MI. (C) Mean duration also significantly diminished (box and whisker plot). n = 9 sham; 11 MI-Veh; 13 MI-T3; # p<0.05 vs. MI-Veh.

The incidence of atrial tachyarrhythmias (ATA) after discontinuation of experimental atrial tachypacing [21] was significantly diminished by 88% (Fig 3B) following T3 (1/13 rats) compared to the vehicle group (7/11 rats). Only two out of nine shams developed arrhythmias. Correspondingly, arrhythmia duration was also significantly attenuated (Fig 3C).

Importantly, anesthetized heart rates (HRs) did not change significantly between the groups (Table 2 and S2 Fig) indicating that T3 is safe and did not induce tachycardia at the dose used. Similarly, the rate-pressure product, an indicator of myocardial oxygen-consumption, remained unchanged following T3 treatment suggesting functional improvements occurred without an increase in metabolic cost. In addition, with anesthetized HR 275–450 bpm, the MI-induced decrease in dP/dtmax was restored following T3 indicating improvement in contractility. Diastolic parameters were not significantly affected.

**Table 2:** Low-dose oral T3 treatment did not adversely affect heart rate and improved contractility.

	Sham	MI	MI+T3
**Heart rate (bpm)**	331±14	324±11	334±8
**Rate-pressure product (mmHg/min)**	39420±2217	35157±1552	36026±1545
**LV dP/dt(max) (mmHg/sec)**	8324±478	6892±228*	7737±285^#^
**LV dP/dt(min) (mmHg/sec)**	8284±656	5119±384***	4967±417***
**LVESP (mmHg)**	117±2	102±5*	99±5*
**LVEDP (mmHg)**	6±0.5	10.6±1.6**	11.9±0.8**
**Tau (msec)**	9.2±0.5	13.3±1.3*	13.4±1.2*
**Arterial BP (mmHg)**	100±2	93±3	96±3

LV–Left ventricular; dP/dt(max)–maximal rate of LV pressure development

dP/dt(min)–maximal rate of LV pressure decline

ESP–End-systolic pressure

EDP–End-diastolic pressure

BP–Blood pressure

T3–triiodo-L-thyronine

MI–Myocardial Infarction

n = 7–8 sham; 7–9 MI-Veh; 11 MI+T3

*p<0.05

**p<0.01

***p<0.001 vs. Sham

^#^p<0.05 vs. MI.

### Decrease in infarct area and length following T3

Histologically, the increase in infarct area following MI-Veh was reduced by 29% in T3-treated MI hearts (Table 3 and and S3 Fig). This was associated with a decrease in infarct length. Furthermore, the viable tissue area in LV mid-section infarct segment partially increased following T3.

**Table 3:** Changes in infarct parameters.

	MI+Veh	MI+T3
**Infarct area (mm**^**2**^**)**	11.9±3.0	8.5±2.9*
**Infarct length (mm)**	10.4±1.4	8.3±2.0*
**Percent infarct area/LV area (%)**	38.2±6.4	32.6±9.8
**Percent viable area within infarct zone (%)**	26.9±16.9	39.7±17.3

LV–Left ventricular

T3–triiodo-L-thyronine

MI–Myocardial Infarction

Veh–Vehicle; means±SD; n = 5–9 MI+Veh; 8 MI+T3

*p = 0.05.

### Oral T3 restores gene expression related to key cardiac and cellular processes

**Fig 4:**
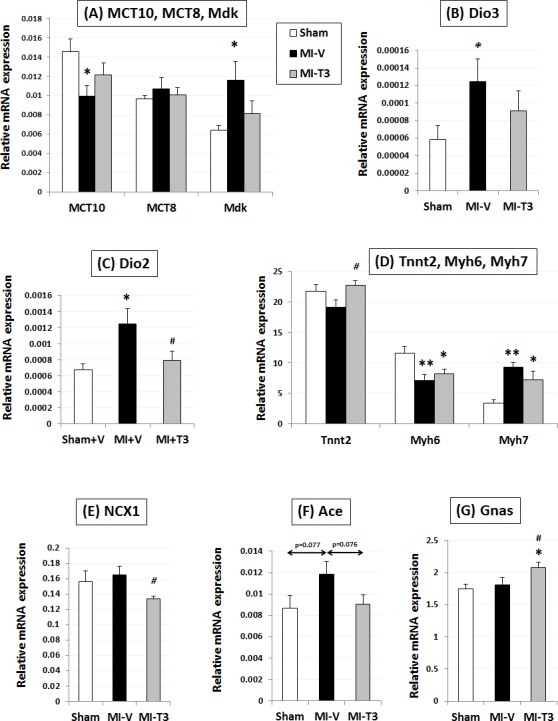
Expression of cardiac genes. Gene expression was normalized using *cyclophilin A* and *Rplp1*. MCT, monocarboxylate transporters; Mdk, midkine; DiO, deiodinase; Tnnt2, cardiac troponin T type 2; Tnni3, cardiac troponin I type 3; Myh, cardiac alpha myosin heavy chains; Ace, angiotensin converting enzyme; NCX1, sodium calcium exchanger 1; Gnas, GNAS complex locus, G protein stimulatory alpha; n = 8 per group; *p<0.05, **p<0.01 vs. Sham; #p<0.05 vs. MI-V (Veh).

Quantitative Real-time PCR using custom array showed that MI significantly impaired the expression levels of thyroid genes including Deiodinase 3 (Dio3) and monocarboxylic acid transporter 10, MCT10 (*Slc16a10*) (Fig 4A–4C and S4 Fig). Oral T3, although not significant, tended to partially improve expression of these genes. Midkine also followed a similar pattern (Fig 4A). Cardiac troponin T (*Tnnt2*) significantly increased and sodium calcium exchanger (NCX1) significantly decreased following oral T3 (Fig 4D and 4E). Beta-myosin heavy chain (*Myh7*) increased 2.9-fold following MI and was reduced to 0.65-fold with T3 (Fig 4D). Sarco-endoplasmic reticulum adenosine triphosphatase and phospholamban levels were not significantly altered (data not shown). Interestingly, the increase (1.38-fold) in angiotensin converting enzyme (*Ace*) levels following MI was downregulated by T3 by 23.4% without significantly altering angiotensin receptors (Fig 4F). In addition, T3 also increased stimulatory G-protein alpha expression (*Gnas*; p<0.05; Fig 4G) without significantly affecting adrenergic receptors or the adenylyl cyclases.

**Fig 5:**
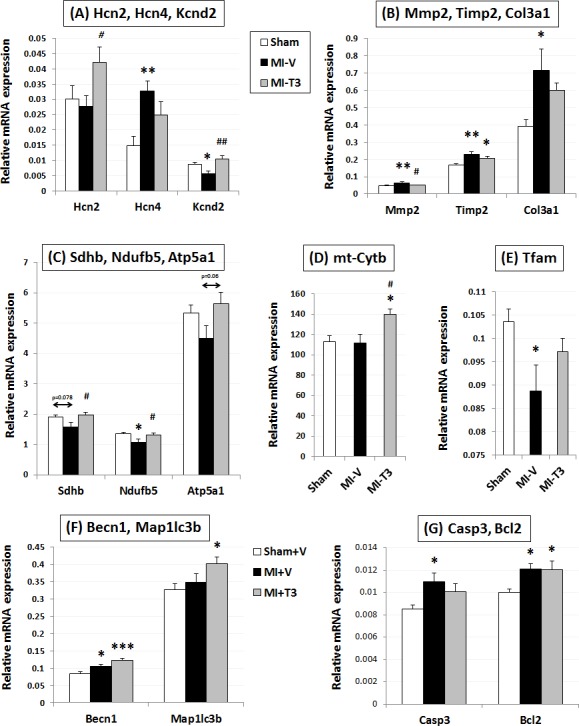
Expression of ion channel, mitochondria-related and extracellular matrix pathway genes. Gene expression was normalized using *cyclophilin A* and *Rplp1*. Hcn, hyperpolarization activated cyclic nucleotide–gated channel; Kcnd2, Potassium voltage-gated channel, Shal-related subfamily, member 2; Mmp2, matrix metallopeptidase 2; Timp2, tissue inhibitor of metalloproteinase 2; Col3a1, Collagen, type III, alpha 1; Sdhb, Succinate dehydrogenase complex, subunit B, iron sulfur (Ip); Ndufb5, NADH dehydrogenase (ubiquinone) 1 beta subcomplex, 5; Atp5a1, ATP synthase, H+ transporting, mitochondrial F1 complex, alpha subunit 1, cardiac muscle; mt-cytb, mitochondrial Cytochrome b; Tfam, Transcription factor A, mitochondrial; Becn1, Beclin 1, autophagy related; Map1lc3b; Microtubule-associated protein 1 light chain 3 beta; Casp3, caspase 3; Bcl2, B-cell CLL/lymphoma 2; n = 8 per group; *p<0.05, **p<0.01 vs. Sham; ***p<0.001 vs. Sham; #p<0.05, ##p<0.01 vs. MI-V (Veh).

THs also restored voltage-gated potassium channel Kv4.2 (*Kcnd2*) and cyclic nucleotide-gated potassium channel *Hcn2* (Fig 5A). The increase in matrix metalloproteinase *Mmp2* levels following MI was prevented by T3 (p<0.05) (Fig 5B). Similarly, the T3-induced reductions in collagens, *Col3a1* and *Col1a1* were not significantly different from the sham levels (Fig 5B and S5 Fig).

Importantly, expression levels of several mitochondrial complex enzymes were restored by T3 (Fig 5C and 5D and S6 Fig). This includes reduced nicotinamide adenine dinucleotide, NADH dehydrogenase (ubiquinone) 1 beta subcomplex, 5 (*Ndufb5*; complex I), succinate dehydrogenase complex, subunit B, iron sulfur (Ip) (*Sdhb*; complex II), mitochondrially encoded cytochrome b (*mt-cytb*; complex III) and adenosine triphosphate, ATP synthase, H+ transporting, mitochondrial F1 complex, alpha subunit 1, cardiac muscle (*Atp5a1*; complex V). Interestingly, T3 further increased expression of Beclin 1 (*Becn1*) and LC3 II (*Map1lc3b*) genes, suggesting an autophagy-mediated novel cardioprotective role in MI (Fig 5F). Proapoptotic caspase 3 (*Casp3*) increased with MI and was not significantly different from sham levels with treatment (Fig 5G). The increase in Glutathione peroxidase 1 (*Gpx1)* was partially restored by T3 (15%) (S6 Fig).

## Discussion

### Low-dose therapeutic T3 in clinical translation

While a number of studies have shown that THs can be safely administered to MI/HF patients [9–11], a general assumption by clinicians and experts, particularly those studying TH analogs, is that maximum benefits may not be achievable within a therapeutic treatment window. This is the first study to show that oral administration of low-dose T3 after acute MI and continued over a 2-month period at doses between 5–8 μg/kg/day (0.05–0.08 μg/ml) significantly improved cardiac structure and function, decreased incidence of tachyarrhythmias, reduced adverse LV remodeling, partially reversed gene reprogramming, and expression of thyroid, mitochondrial, neurohumoral, sarcomeric, ion channel and fibrotic genes. All of these were accomplished without significant negative changes in HR, rate-pressure product, serum TH concentrations or heart weight indicating safe cardiovascular therapeutic benefits. This is the 3^rd^ paper in a series of experiments from our lab exploring therapeutic oral T3 treatment benefits in heart diseases. Previous studies in rats with diabetic cardiomyopathy and hypertension [5, 14] demonstrated significant cardioprotective benefits with low-dose oral T3 therapy at 3–4 μg/kg/day (0.03–0.04 μg/ml). We tested doses ranging from 3–8 μg/kg/day in 2 additional cohorts of infarct rats (unpublished) and found that doses exceeding 8 μg/kg/day led to a borderline increase in HR and/or decreasing serum tT4/TSH levels (expected feedback response). Taken together, cumulative data from our lab now suggest that oral T3 doses between 3–8 μg/kg/day are safe and effective in treating cardiovascular disorders in rats.

Based on our observations, rats subjected to MI surgeries take ~6 hours to start drinking water. In spite of this delay, remarkable benefits in cardiac structure/function, rhythm, remodeling, etc. were observed. This finding suggests possible cardiac benefits in patients who are unable to receive rapid reperfusion therapy. Short-term TH treatment inhibited myocyte apoptosis during early post-MI remodeling [15], and improved myocyte preservation in the peri-infarct zone after long-term T4 treatment [8, 18]. Additional mechanistic studies exploring the protective mechanism of acute oral T3 are warranted. The non-infarct anatomical and isolated myocyte shape pattern after MI has been shown to be virtually identical in rats and humans. Consequently, there is good reason for optimism about translation from the current studies to humans. Unlike patients with MI, these rats were subjected only to experimental ischemic injury and did not have preexisting/associated risk factors like atherosclerosis that can worsen the prognosis. However, the inhibitory effect of THs on serum lipids and atherosclerosis may offer further benefits in humans [23, 24]. Free T3, with its low serum protein binding properties, is now recognized as an important biomarker in HF and our data also supports these clinical observations [4, 25, 26].

### THs and cardiac remodeling

Oral T3 significantly improved or partially normalized expression of fetal genes including beta-myosin heavy chain, alpha-myosin heavy chain, cardiac troponin T and sodium calcium exchanger [1, 7, 8]. G-protein stimulatory alpha is an important mediator of beta-adrenergic signaling, and its mRNA expression/activity is compromised in hypertrophy and HF. Although we did not find such a decrease following MI, low-dose oral T3 increased Gs alpha expression indicating potential therapeutic effects [1]. In addition, the increase in post-MI ACE expression was also partially decreased with T3, indicating potential cardioprotective effects via reducing the sympathetic neural activation characteristic of MI/HF [27]. Similar to our past observations [5, 18, 28], we also found significant attenuation in infarct fibrotic area and associated restoration in MMP and collagen gene expression. We have already published several studies [6, 7, 15, 29] on acute T3/T4 cardioprotective effects and hence did not repeat them.

### THs and arrhythmia

We recently demonstrated that ATA are inducible in post-MI [30] and hypothyroid [21] rats that have cardiac dysfunction as suggested by some clinical observations [6, 26]. T4 treatment reduced ATA inducibility post-MI with associated attenuation in atrial fibrosis. In the present study, we have, for the first time shown significant attenuation/prevention of arrhythmias with therapeutic low-dose oral T3 treatment post-MI without a change in HR, indicating remarkable clinical potential. Mechanistically, this may be partially due to effects on ion channel gene expression observed here (Kv4.2 and *Hcn2*) [1, 31, 32]. Besides genomic mechanisms, THs act via nongenomic signaling [2, 33]. Therefore, acute treatment studies will be helpful to dissect these mechanisms. Considering that a significant portion of cardiac deaths are linked to arrhythmias, this issue certainly merits clinical investigation [34].

### Mechanisms of therapeutic low-dose oral T3-mediated cardioprotection

Mitochondria are critical for cardiac energy homeostasis and THs are known to affect mitochondrial biogenesis and function via classical nuclear receptor-mediated or direct mitochondrial mechanisms [6, 35, 36]. We found that therapeutic T3 treatment restored expression of genes from the mitochondrial respiratory chain complexes I, II, III and V–both nuclear- and mitochondrial-encoded. Autophagy has been considered to be protective or detrimental depending on a number of factors including type or duration of injury [37, 38]. However, the role of THs in mediating cardiac autophagy is unclear. Recent studies indicate T3-mediated stimulation of autophagy in liver and skeletal muscle [36]. This is the first known study to demonstrate that low-dose oral T3 further increased Beclin-1 and LC3 II expression indicating a possible protective role of cardiac autophagy 2-mo post-MI. It is now a well-understood phenomenon that animal models of heart diseases, such as ischemia [39–41], diabetic cardiomyopathy [5] and hypertensive/hypertrophic HF [14, 40, 42] result in increased Dio3, decreased MCT-10 (one of the important T3 transporters) along with fetal gene reexpression. A number of studies from our laboratory have shown that LV functional improvement and altered Dio3, MCT10 and fetal genes are intimately linked to each other in DM, hypertension, or hypothyroidism and TH treatment of rats reverse these abnormalities [5, 14, 17, 43]. The findings in the current study reinforces all these mechanisms and T3 partially restored them.

### Limitations

The limitations of the study include the following. It remains to be shown whether the improved cardiac structure/function and reduced tachyarrhythmia are associated with favorable long-term survival. Female rats were used in the study since, unlike males, they maintain a more stable body mass during altered thyroid states. Compared to males, females also have a much higher incidence of thyroid disorders associated with coronary artery disease [23]. In addition, we have previously shown that post-MI changes in LV function and myocyte remodeling in similar young adult Sprague-Dawley rats are remarkably similar in male and female rats [44]. Other studies using males have shown cardioprotective benefits [8, 45] and the selection of females only is not likely to affect generalization of the results to male rats. Nevertheless, further investigation would be helpful. Multiple sections of infarcted LV were not acquired for assessment of infarct characteristics since we have repeatedly shown transverse mid wall LV sections to be representative of the LV, which is consistent with the observations of others [38, 46]. Our experience from several hundreds of MI microsurgeries has shown remarkable consistency. For instance, the mean infarct length of 2 months post-MI is virtually the same number in about a dozen repeat experiments over the years. Any small infarction is excluded by early screening. A sham T3 treatment group was not deemed necessary since the focus of the study was therapeutic treatment of MI. The pharmacology of TH treatment of normal rats (hyperthyroidism) has been extensively reported.

## Conclusions

MI, like other cardiac pathologies [14, 40–42], is associated with Dio3 upregulation and fetal gene re-expression, impairments in cardiac structure, function and rhythm. This is the first study to establish a safe therapeutic window using low-dose enteral/oral T3, providing significant therapeutic benefits over the aforementioned defects without adverse effects on the cardiovascular system. These changes are likely not due to hemodynamic/systemic effects as neither arterial pressure nor serum thyroid levels were significantly altered. Based on our gene expression results and numerous published *in vitro/ex vivo* studies from our and other groups, these benefits likely stem from TH effects on local actions [6, 7, 29].

As seen in our studies, feedback inhibition of tT4 and TSH provide a reasonable guide to monitor serum T3 levels with remarkable cardiovascular benefits. Given the upregulation of Dio3 and subsequent conversion of T4 to inactive rT3, it seems logical to use T3, the active form of TH for therapy. Nonetheless, we have observed similar benefits with T4 [18, 30, 43], as have many clinical studies. The issue of T3, T4, or both as the ideal treatment mode merits further investigation. As is the case with T4 (Synthroid) or T3/T4 (Armour), T3 is commercially available, inexpensive, and can therefore offer remarkable healthcare savings. Preliminary data from the THiRST PONTE pilot study suggests safe therapeutic benefits with similar T3 protocol in acute MI patients. However, longer-term randomized, placebo-controlled large clinical trials are awaited.

## Supporting Information

S1 Fig Cardiac Magnetic Resonance Imaging (MRI). Representative Cardiac MRI from mid-ventricular short axis showing systolic and diastolic phases of the three groups at 2 mo. post-op. Arrowheads point to dyssynergic infarct area. In a triiodo-L-thyronine (T3)-treated heart, the infarct area is reduced.
(TIF)

S2 Fig Pressure tracings. Representative left ventricular pressure tracings.
(TIF)

S3 Fig Cardiac histology. Representative transverse mid-left ventricular 2x histological sections following trichrome staining showing partially attenuated infarct characteristics with oral T3 treatment.
(TIF)

S4 Fig Expression of cardiac genes. Gene expression was normalized using *cyclophilin A* and *Rplp1*. Thrb, thyroid hormone receptor beta; Nppb, natriuretic peptide precursor B; Tnni3, cardiac troponin I type 3; n = 8 per group; **p<0.01 vs. Sham.
(TIF)

S5 Fig Expression of extracellular matrix pathway genes. Gene expression was normalized using *cyclophilin A* and *Rplp1*. Col1a1, Collagen, type I, alpha 1; Tgfb, transforming growth factor beta; n = 8 per group; *p<0.05 vs. Sham.
(TIF)

S6 Fig Expression of mitochondria-based pathway genes. Gene expression was normalized using *cyclophilin A* and *Rplp1*. Ndufa5, NADH dehydrogenase (ubiquinone) 1 alpha subcomplex 5; Nox4, NADPH oxidase 4; Ppargc1a, Peroxisome proliferator-activated receptor gamma, coactivator 1 alpha; Hspa9, Heat shock protein 9; Gpx1, Glutathione peroxidase 1; n = 8 per group; *p<0.05, **p<0.01 vs. Sham.
(TIF)

S1 Table Primers used for gene expression experiments. Primers were obtained from SABiosciences (Qiagen Inc., Valencia, CA). The RefSeq Accession number refers to the representative sequence used to design the enclosed primers.
(DOCX)
