# Supplementary figures and images for: Safe Oral Triiodo-L-Thyronine Therapy Protects from Post-Infarct Cardiac Dysfunction and Arrhythmias without Cardiovascular Adverse Effects
Source: PLoS One. 2016 Mar 16;11(3):e0151413. doi: 10.1371/journal.pone.0151413 (PMC4794221; doi:10.1371/journal.pone.0151413)

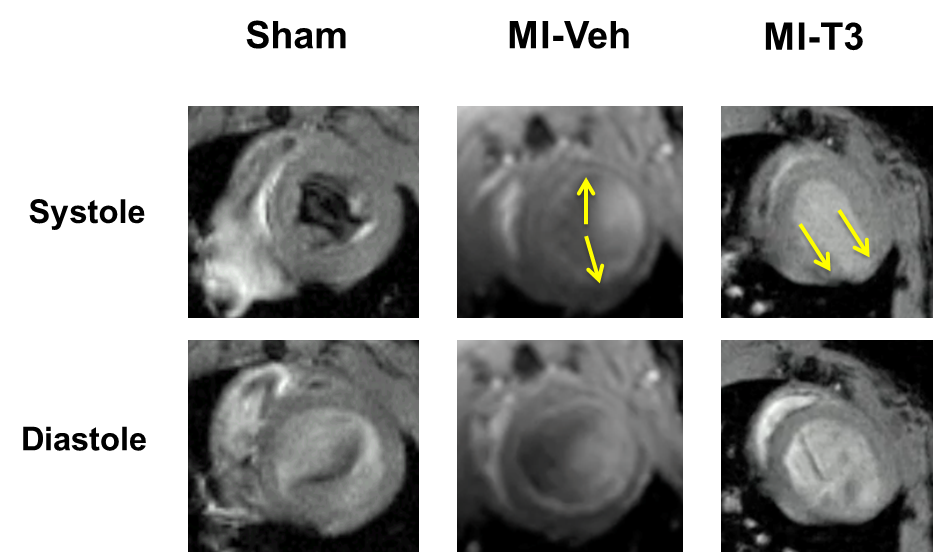

Supplement: S1 Fig — Representative Cardiac MRI from mid-ventricular short axis showing systolic and diastolic phases of the three groups at 2 mo. post-op. Arrowheads point to dyssynergic infarct area. In a triiodo-L-thyronine (T3)-treated heart, the infarct area is reduced. (TIF) [file pone.0151413.s001.tif]

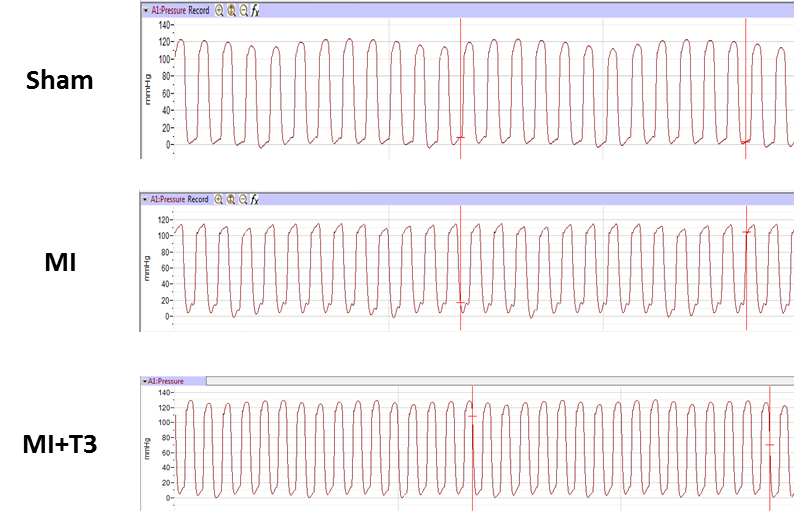

Supplement: S2 Fig — Representative left ventricular pressure tracings. (TIF) [file pone.0151413.s002.tif]

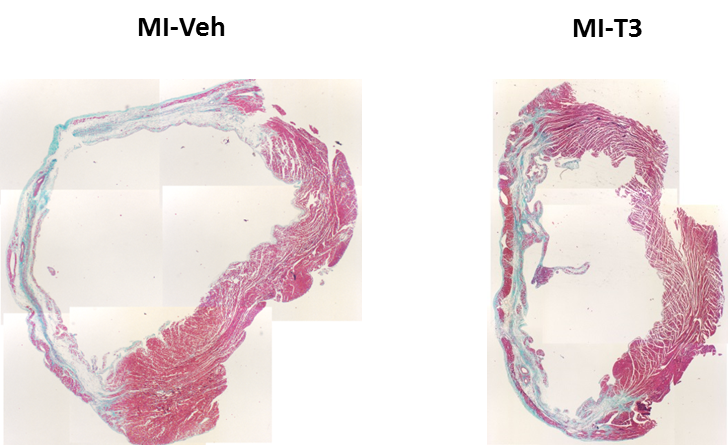

Supplement: S3 Fig — Representative transverse mid-left ventricular 2x histological sections following trichrome staining showing partially attenuated infarct characteristics with oral T3 treatment. (TIF) [file pone.0151413.s003.tif]

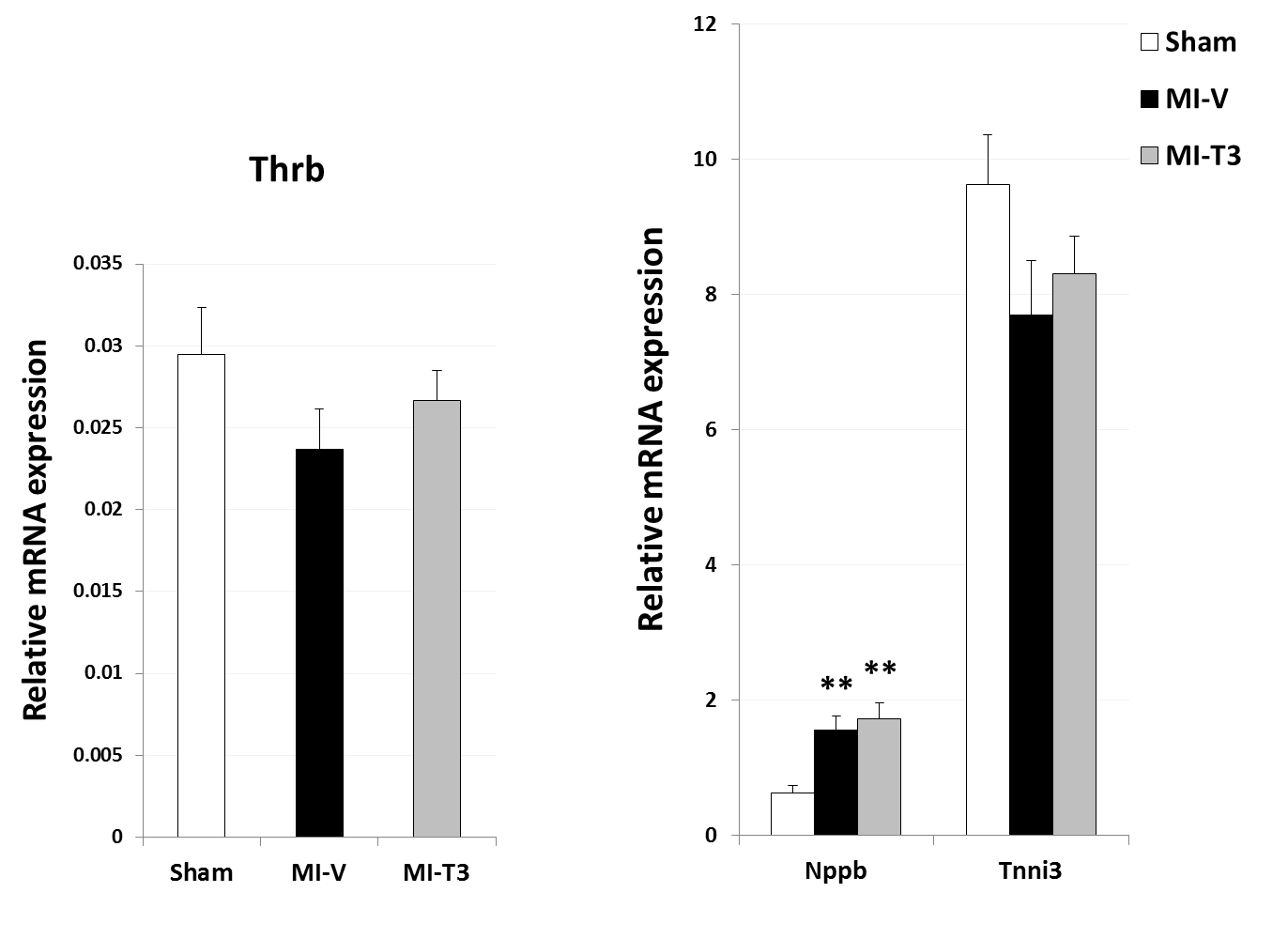

Supplement: S4 Fig — Gene expression was normalized using cyclophilin A and Rplp1. Thrb, thyroid hormone receptor beta; Nppb, natriuretic peptide precursor B; Tnni3, cardiac troponin I type 3; n = 8 per group; **p<0.01 vs. Sham. (TIF) [file pone.0151413.s004.tif]

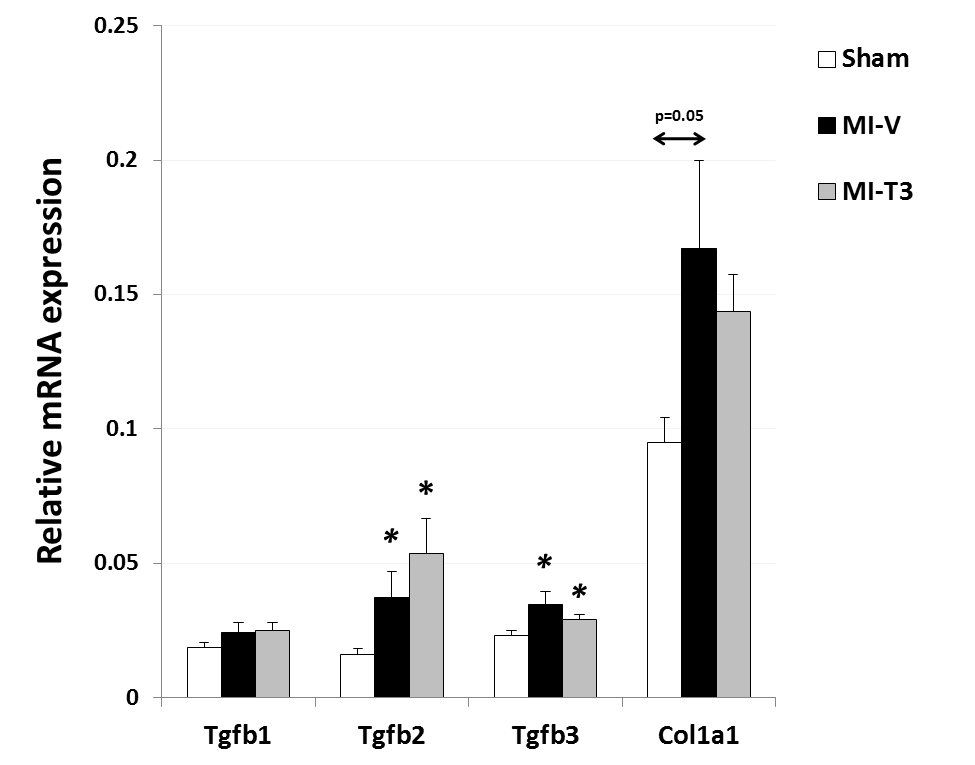

Supplement: S5 Fig — Gene expression was normalized using cyclophilin A and Rplp1. Col1a1, Collagen, type I, alpha 1; Tgfb, transforming growth factor beta; n = 8 per group; *p<0.05 vs. Sham. (TIF) [file pone.0151413.s005.tif]

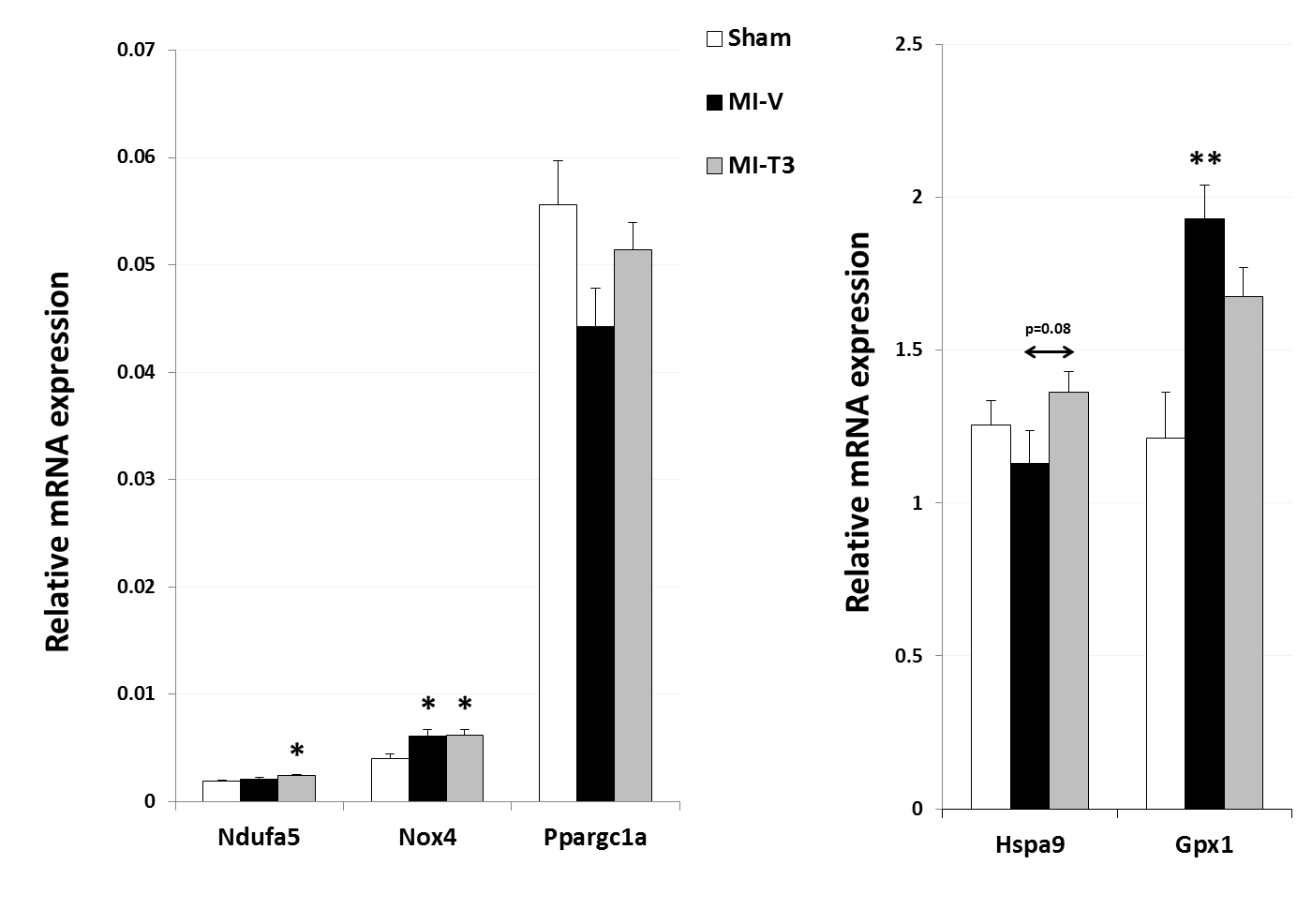

Supplement: S6 Fig — Gene expression was normalized using cyclophilin A and Rplp1. Ndufa5, NADH dehydrogenase (ubiquinone) 1 alpha subcomplex 5; Nox4, NADPH oxidase 4; Ppargc1a, Peroxisome proliferator-activated receptor gamma, coactivator 1 alpha; Hspa9, Heat shock protein 9; Gpx1, Glutathione peroxidase 1; n = 8 per group; *p<0.05, **p<0.01 vs. Sham. (TIF) [file pone.0151413.s006.tif]
