# Supplementary material for: Safe Oral Triiodo-L-Thyronine Therapy Protects from Post-Infarct Cardiac Dysfunction and Arrhythmias without Cardiovascular Adverse Effects
Source: PLoS One. 2016 Mar 16;11(3):e0151413. doi: 10.1371/journal.pone.0151413 (PMC4794221; doi:10.1371/journal.pone.0151413)
Supplement: S1 Table — Primers were obtained from SABiosciences (Qiagen Inc., Valencia, CA). The RefSeq Accession number refers to the representative sequence used to design the enclosed primers. (DOCX) [file pone.0151413.s007.docx]

**SUPPORTING INFORMATION**

**S1 Table.**

| **Description** | **Gene Name** | **Symbol** | **RefSeq** |
| --- | --- | --- | --- |
| Thyroid hormone receptor beta | C-erba-beta/ERBA2/Nr1a2/RATT3REC/T3rec/TRbeta | Thrb | NM_012672 |
| Thyroid hormone receptor alpha | ERBA1/Thra1 | Thra | NM_031134 |
| Deiodinase, iodothyronine, type III | 5DIII/DIOIII | Dio3 | NM_017210 |
| Deiodinase, iodothyronine, type II | 5DII/DIOII | Dio2 | NM_031720 |
| Deiodinase, iodothyronine, type I | 5DI/ITDI1/MGC93462/TXDI1 | Dio1 | NM_021653 |
| Midkine | - | Mdk | NM_030859 |
| Crystallin, mu | MGC108623 | Crym | NM_053955 |
| Solute carrier family 16, member 2 (monocarboxylic acid transporter 8) | - | Slc16a2 | NM_147216 |
| Solute carrier family 16 (monocarboxylic acid transporters), member 10 | Tat1 | Slc16a10 | NM_138831 |
| ATPase, Ca++ transporting, cardiac muscle, slow twitch 2 | Serca2/SercaII | Atp2a2 | NM_001110139 |
| Myosin, heavy chain 6, cardiac muscle, alpha | Myhca | Myh6 | NM_017239 |
| Myosin, heavy chain 7, cardiac muscle, beta | Myhcb | Myh7 | NM_017240 |
| Phospholamban | Plm | Pln | NM_022707 |
| Troponin I type 3 (cardiac) | TnI/cTNI | Tnni3 | NM_017144 |
| Troponin T type 2 (cardiac) | CTTG/Ctt/RATCTTG/Tnnt3 | Tnnt2 | NM_012676 |
| ATPase, Na+/K+ transporting, alpha 1 polypeptide | Nkaa1b | Atp1a1 | NM_012504 |
| ATPase, Na+/K+ transporting, beta 2 polypeptide | ATPB2/ATPB2S/Amog/MGC93648/RATATPB2S | Atp1b2 | NM_012507 |
| Solute carrier family 8 (sodium/calcium exchanger), member 1 | Ncx/Ncx1 | Slc8a1 | NM_019268 |
| Ryanodine receptor 2, cardiac | RyR | Ryr2 | NM_001191043 |
| Collagen, type I, alpha 1 | COLIA1 | Col1a1 | NM_053304 |
| Collagen, type III, alpha 1 | MGC93704 | Col3a1 | NM_032085 |
| Transforming growth factor, beta 1 | - | Tgfb1 | NM_021578 |
| Transforming growth factor, beta 2 | - | Tgfb2 | NM_031131 |
| Transforming growth factor, beta 3 | MGC105479 | Tgfb3 | NM_013174 |
| Matrix metallopeptidase 1a (interstitial collagenase) | - | Mmp1a | NM_001134530 |
| Matrix metallopeptidase 2 | - | Mmp2 | NM_031054 |
| TIMP metallopeptidase inhibitor 2 | MGC105282 | Timp2 | NM_021989 |
| Hypoxia-inducible factor 1, alpha subunit (basic helix-loop-helix transcription factor) | MOP1 | Hif1a | NM_024359 |
| Vascular endothelial growth factor A | VEGF164/Vegf | Vegfa | NM_031836 |
| Nitric oxide synthase 3, endothelial cell | eNos | Nos3 | NM_021838 |
| Nitric oxide synthase 2, inducible | Nos2a/iNos | Nos2 | NM_012611 |
| Natriuretic peptide precursor B | BNP/Bnf | Nppb | NM_031545 |
| Natriuretic peptide receptor A/guanylate cyclase A (atrionatriuretic peptide receptor A) | Anpra/Gca/NPR-A/Npra | Npr1 | NM_012613 |
| Cholinergic receptor, nicotinic, alpha 7 | BTX/NARAD/nAChRa7 | Chrna7 | NM_012832 |
| Angiotensin II receptor, type 1a | AT1/AT1A/AT1R/Agtr1 | Agtr1a | NM_030985 |
| Angiotensin II receptor, type 2 | AT2-R/AT2R/AT<sub>2</sub>R | Agtr2 | NM_012494 |
| Angiotensin I converting enzyme (peptidyl-dipeptidase A) 1 | Dcp1/StsRR92 | Ace | NM_012544 |
| Renin | RATRENAA/RENAA/Ren1 | Ren | NM_012642 |
| Adrenergic, alpha-1A-, receptor | Adra1c | Adra1a | NM_017191 |
| Adrenergic, alpha-1B-, receptor | - | Adra1b | NM_016991 |
| Adrenergic, beta-1-, receptor | B1AR/RATB1AR | Adrb1 | NM_012701 |
| Adrenergic, beta-2-, receptor, surface | - | Adrb2 | NM_012492 |
| Adrenergic, beta-3-, receptor | ADRB | Adrb3 | NM_013108 |
| Adenylate cyclase 5 | - | Adcy5 | NM_022600 |
| Adenylate cyclase 6 | ACVI/ADCYB | Adcy6 | NM_012821 |
| Guanine nucleotide binding protein (G protein), alpha inhibiting 1 | BPGTPB | Gnai1 | NM_013145 |
| GNAS complex locus | Gnas1/Gnpas/Nesp55 | Gnas | NM_019132 |
| 3-hydroxy-3-methylglutaryl-Coenzyme A reductase | 3H3M | Hmgcr | NM_013134 |
| Tumor necrosis factor (TNF superfamily, member 2) | MGC124630/RATTNF/TNF-alpha/Tnfa | Tnf | NM_012675 |
| Interleukin 6 | ILg6/Ifnb2 | Il6 | NM_012589 |
| Interleukin 33 | RGD1311155 | Il33 | NM_001014166 |
| Interleukin 1 beta | - | Il1b | NM_031512 |
| Interleukin 10 | IL10X | Il10 | NM_012854 |
| Interferon regulatory factor 7 | MGC125013 | Irf7 | NM_001033691 |
| Actin, alpha, cardiac muscle 1 | MGC156490 | Actc1 | NM_019183 |
| Actin, alpha 1, skeletal muscle | - | Acta1 | NM_019212 |
| Solute carrier family 2 (facilitated glucose transporter), member 4 | Glut4/MGC93607 | Slc2a4 | NM_012751 |
| Superoxide dismutase 2, mitochondrial | - | Sod2 | NM_017051 |
| Catalase | CS1/Cas1/Cs-1/RATCAT01/RATCATL | Cat | NM_012520 |
| NADPH oxidase 4 | - | Nox4 | NM_053524 |
| Glutathione peroxidase 1 | GSHPx/GSHPx-1 | Gpx1 | NM_030826 |
| Malic enzyme 1, NADP(+)-dependent, cytosolic | MOD1 | Me1 | NM_012600 |
| Beclin 1, autophagy related | - | Becn1 | NM_053739 |
| ATG5 autophagy related 5 homolog (S. cerevisiae) | - | Atg5 | NM_001014250 |
| Microtubule-associated protein 1 light chain 3 beta | MGC93422/Map1lc3/Mpl3/zbs559 | Map1lc3b | NM_022867 |
| Protein kinase, AMP-activated, alpha 2 catalytic subunit | AMPK | Prkaa2 | NM_023991 |
| Mechanistic target of rapamycin (serine/threonine kinase) | Frap1/RAFT1 | Mtor | NM_019906 |
| NADH dehydrogenase (ubiquinone) 1 alpha subcomplex 5 | MGC72911/NADHUO | Ndufa5 | NM_012985 |
| NADH dehydrogenase (ubiquinone) 1 beta subcomplex, 5 | - | Ndufb5 | NM_001106426 |
| Cytochrome c oxidase, subunit Va | - | Cox5a | NM_145783 |
| Succinate dehydrogenase complex, subunit B, iron sulfur (Ip) | - | Sdhb | NM_001100539 |
| Cytochrome b, mitochondrial | mt-Cytb | mt-Cytb | YP_665641 |
| ATP synthase, H+ transporting, mitochondrial F1 complex, alpha subunit 1, cardiac muscle | - | Atp5a1 | NM_023093 |
| Heat shock protein 9 | Hspa9a | Hspa9 | NM_001100658 |
| Bcl2-associated X protein | - | Bax | NM_017059 |
| B-cell CLL/lymphoma 2 | Bcl-2 | Bcl2 | NM_016993 |
| Caspase 3 | Lice/MGC93645 | Casp3 | NM_012922 |
| NK2 transcription factor related, locus 5 (Drosophila) | Csx/Nkx2.5 | Nkx2-5 | NM_053651 |
| Ubiquitin B | UBC | Ubb | NM_138895 |
| Peroxisome proliferator-activated receptor gamma, coactivator 1 alpha | Ppargc1 | Ppargc1a | NM_031347 |
| Transcription factor A, mitochondrial | Mttfa | Tfam | NM_031326 |
| Fibroblast growth factor 2 | Fgf-2/bFGF | Fgf2 | NM_019305 |
| Calcium channel, voltage-dependent, L type, alpha 1C subunit | RATIVS302 | Cacna1c | NM_012517 |
| Hyperpolarization activated cyclic nucleotide-gated potassium channel 2 | - | Hcn2 | NM_053684 |
| Hyperpolarization activated cyclic nucleotide-gated potassium channel 4 | - | Hcn4 | NM_021658 |
| Potassium voltage-gated channel, shaker-related subfamily, member 5 | Kv1/Kv1.5 | Kcna5 | NM_012972 |
| Potassium voltage-gated channel, Shal-related subfamily, member 2 | Kv4.2/RK5/Shal1 | Kcnd2 | NM_031730 |
| Mitogen activated protein kinase 14 | CRK1/CSBP/CSPB1/Csbp1/Csbp2/Exip/Hog/MGC105413/Mxi2/Prkm14/Prkm15/RK/Sapk2A/p38/p38Hog/p38alpha | Mapk14 | NM_031020 |
| V-akt murine thymoma viral oncogene homolog 1 | Akt | Akt1 | NM_033230 |
| Peptidylprolyl isomerase A (cyclophilin A) | CYCA/MGC72881 | Ppia | NM_017101 |
| Ribosomal protein, large, P1 | MGC72935 | Rplp1 | NM_001007604 |
